# Parent discrimination clusters and pediatric health in a national survey: The modifying effect of parenting

**DOI:** 10.1016/j.ssmph.2025.101757

**Published:** 2025-01-25

**Authors:** Violeta J. Rodriguez, Dominique L. La Barrie

**Affiliations:** a Department of Psychology, University of Illinois Urbana Champaign, Champaign, IL, 61820, United States; b Department of Psychology, University of Georgia, Athens, Georgia, United States

## Abstract

• The study uses unsupervised machine learning to identify discrimination clusters in parents.
• Four distinct clusters of parental discrimination were identified using machine learning.
• Parental discrimination clusters are associated with poorer pediatric health outcomes.
• Positive parenting may mitigate the negative effects of parent discrimination on child health.
• Negative parenting may exacerbate the health risks associated with parental discrimination.

Individuals from ethnoracially minoritized groups frequently endure discrimination, manifesting as unfair treatment, suspicion, and ridicule (Carroll, 1998). This pervasive discrimination, which may entail diminished respect, and being treated poorly or as inferior, has been reported by 50–70% of Black, Hispanic, and Asian individuals in the United States (Lee et al., 2019) making it a significant and chronic stressor for these communities. Various theoretical models suggest that discrimination's pervasive nature ultimately impacts not only the individuals who experience it firsthand but can extend to the family unit, including family relationships and parenting behaviors (Carroll, 1998; Coll et al., 1996; McNeil Smith & Landor, 2018). In turn, these dynamics relate to various aspects of youth development. Moreover, parental experiences of discrimination have been shown to predict worse child mental health (Tran, 2014), and also have been shown to influence parenting (Anderson et al., 2015; Zong, Cheah, Ren, & Hart, 2023).

The sociocultural family stress model (McNeil Smith & Landor, 2018) suggests that discrimination, as a chronic stressor, can disrupt family functioning and amplify negative parenting behaviors, which in turn negatively affect child outcomes. This model aligns with the broader literature indicating that parental discrimination experiences are associated with poorer relational dynamics and family stress (Armah et al., 2024; Ayón & García, 2019; Condon et al., 2022; Holloway & Varner, 2021; Lavner et al., 2018; Rice et al., 2023; Zong, Cheah, Ren, & Hart, 2023). Further, the mundane extreme environmental stress model (Carroll, 1998) conceptualizes discrimination as a persistent environmental stressor that influences health outcomes across generations. This framework highlights the multifaceted ways in which discrimination impacts families, including both the direct effects of stress on health as well as the role that family relationships and dynamics, such as parenting, can play in modifying these associations. Together, these models emphasize the importance of examining parenting as a moderating factor that shapes the association between discrimination and child health. This growing evidence highlights the need to further investigate how parenting can either buffer or exacerbate the impact of discrimination on children's mental and physical health. Indeed, in recent decades, significant strides have been made in understanding the complex interplay of factors contributing to health disparities, particularly in maternal and child health. Central to this understanding is the recognition that discrimination—whether based on race, ethnicity, or socioeconomic status—profoundly affects health outcomes not only for the individuals directly experiencing it but also for their children.

The Preconception Stress and Resiliency Pathways (PSRP) Model also provides a comprehensive framework for understanding the multifaceted influences on maternal, paternal, and child health (Ramey et al., 2015). Developed through community-based participatory research, this model emphasizes that parental stress and resilience can have profound impacts on health outcomes across generations. Discrimination, as a unique stressor, can therefore trigger a cascade of biological responses in parents, known as allostatic load, which comprises the physiological wear and tear associated with chronic stress. This heightened allostatic load during the preconception period can adversely affect reproductive health and, by extension, child health outcomes from conception onwards. Moreover, the PSRP model articulates how parental resilience can mitigate or exacerbate the effects of stress. For instance, positive parenting—such as nurturing, supportive, and responsive caregiving—can buffer children from the deleterious effects of discrimination by promoting better emotional regulation and stress resilience. Conversely, negative parenting, which may be more prevalent under conditions of high stress and discrimination, can impair a child's ability to cope with stress, leading to poorer physical and mental health outcomes.

While previous research has focused on the detrimental effects of negative parenting in the context of parental discrimination (Anderson et al., 2015; Savell et al., 2024), there remains a significant gap in understanding how both negative *and* positive parenting influences children's health outcomes in the context of discrimination. Specifically, there is a need to better understand whether positive parenting consistently buffers the adverse effects of discrimination or whether its efficacy varies across contexts. Likewise, the role of negative parenting in amplifying the harmful impacts of discrimination remains understudied. Moreover, existing studies do not differentiate between positive and negative parenting, potentially overlooking how discrimination may affect *both* (Condon et al., 2022). Additionally, much of the existing literature is limited by small sample sizes, a predominant focus on African American populations, and mental health (Anderson et al., 2015; Condon et al., 2022), limiting our understanding of other pediatric outcomes, such as physical functioning, which may also be impacted by discrimination (Williamson et al., 2023).

The effect of discrimination on pediatric health is a critical concern, especially for ethnoracially minoritized groups. Studies have shown that parents' experiences of discrimination can have adverse effects on children's health (Anderson et al., 2015; Zong, Cheah, Ren, & Hart, 2023). These adverse effects include increased symptoms of anxiety, depression, and other stress-related conditions, as well as potential impacts on physical health, such as sleep disturbances and somatic complaints. Positive parenting practices may buffer children against these harmful impacts, while negative parenting behaviors may exacerbate them, amplifying the stress of discrimination. Understanding how these moderating effects vary across health domains—both mental and physical—is essential for identifying effective interventions.

Our study builds on these theoretical frameworks to test how parenting behaviors moderate the association between discrimination clusters and pediatric health. By integrating unsupervised machine learning methods to identify discrimination clusters, we aim to better understand how discrimination patterns (e.g., frequency and attributions) interact with parenting practices to shape health. To address these gaps in the literature, our study uses an unsupervised machine learning method, sidClustering (Ishwaran, Mantero, et al., 2023; Mantero & Ishwaran, 2021), to identify patterns of discrimination experiences and attributions (e.g., perceived reasons for discrimination) in an ethnoracially diverse cohort of parents. By isolating clusters, we aim to capture the multifaceted nature of discrimination experiences beyond the linear assessments of frequency continuous scores. Clustering provides a way to identify and categorize distinct patterns of discrimination experiences that might be lost when using continuous measures. Isolating clusters of discrimination experiences, as opposed to relying solely on continuous scores, provides the benefits of capturing potentially complex patterns, offering a better understanding, potentially enabling tailored interventions (e.g., which clusters of people are at greater risk), enhancing interpretability, and in this study, identifying interactions between discrimination and parenting on pediatric health.

Clustering approaches, such as sidClustering, have been used to examine how discrimination frequency and attributions relate to health outcomes, revealing patterns missed by traditional methods (Cobb et al., 2023). Unsupervised machine learning techniques like sidClustering are particularly adept at handling complex, high-dimensional data, allowing for more accurate and generalizable insights into the multifaceted nature of discrimination and its impact on health, ultimately informing targeted interventions for at-risk families. Having identified discrimination clusters, we then examine the relations between these clusters and the parent-reported Patient Reported Outcomes Measurement Information System (PROMIS) parent proxy report of pediatric health outcomes, specifically mental (e.g., symptoms of anxiety, depression) and physical (e.g., mobility, sleep, fatigue, and pain) health. Additionally, we investigate the moderating effects of positive and negative parenting on these associations. Our objectives and hypotheses are formulated to explore the clustering of discrimination experiences and their implications for pediatric health.

## Hypotheses

Our hypotheses have been preregistered on the OSF (see Data Availability). Based on a previous study using clustering methods to analyze experiences of discrimination and attributions (Cobb et al., 2023), we hypothesized four clusters would emerge, including (1) low levels of discrimination with few attributions (i.e., reasons or causes that individuals assign to their experiences of discrimination), reflecting sporadic encounters, (2) moderate frequencies of discrimination with attributions primarily related to visible characteristics such as race and gender, (3) high frequencies of discrimination with a wide range of attributions, indicating pervasive discrimination across multiple domains, and (4) very high frequencies of discrimination with extensive attributions, signifying an acute and multifaceted experience of discrimination. Regarding pediatric health outcomes, we draw our hypothesis from previous theoretical models (Carroll, 1998; Coll et al., 1996; McNeil Smith & Landor, 2018) and predict that as the frequency and diversity of discrimination attributions increase across clusters, there will be a corresponding deterioration in pediatric health. Specifically, the cluster experiencing the most frequent discrimination and numerous attributions is hypothesized to exhibit the poorest health, characterized by higher reports of both mental and physical health outcomes. Lastly, we hypothesize that negative parenting will exacerbate the adverse health effects of discrimination, magnifying the associations between discrimination and pediatric health. Conversely, positive parenting is expected to serve as a buffer, mitigating associations between discrimination and pediatric health. Thus, this study examines the interplay between parental discrimination and parenting, and how this complex association may influence pediatric health outcomes within diverse ethnoracial contexts. Findings may contribute to the development of more effective support interventions for families navigating the chronic stress of discrimination.

## 1 Method

### 2 Ethical approval

Ethical approval from the University of Illinois Urbana Champaign Institutional Review Board was obtained.

### 2.1 Participant eligibility

To qualify for the survey, parents had to be 18 years or older, actively parenting at least one child between the ages of 3–17, and cohabitating with the child(ren) for at least half of the time. When a parent had multiple children, they were to focus on the child whose birthday occurred most recently. Only one parent per household was allowed to participate to ensure data independence.

### 2.2 National survey

The survey was conducted nationwide using the services of QualtricsXM, a firm specializing in data gathering and analysis. QualtricsXM recruits from a pool of adults across the nation willing to partake in surveys for compensation. Qualtrics screened their panels for suitability and then sent eligible participants an individualized link to the survey. Upon completion, participants received monetary compensation or compensation of their choice (e.g., money, airline miles). The survey was open from January to February 2024 and was only offered in English. This included a preliminary phase with 150 participants to determine an average completion time for the survey. Responses from participants completing the survey significantly quicker than 1.5 standard deviations below this average were discarded. Though *n* = 1509 participants met criteria and completed the survey, to ensure data integrity, only responses from participants who passed specific attention and quality control questions were considered (*n* = 1444). While we oversampled for ethnoracial minorities in the current sample, other sociodemographic characteristics reflect the sociodemographic of the latest United States Census.

### 2.3 Measures

**Demographic Information.** Participants provided details on various sociodemographic factors, including age, gender, race, ethnicity, education, relationship status, employment status, main sources of income, as well as personal and family income. Information on their living situation, specifically the amount of time spent with their child(ren), was also collected.

**Everyday Discrimination Scale.** Study participants completed the Everyday Discrimination Scale (Williams et al., 1997), which measures the frequency of discrimination encounters through ten items on a five-point scale, with responses ranging from five for "almost every day" to zero for "never." Examples of items on the scale include instances where participants felt they were treated with less courtesy or respect, received inferior service at establishments, were perceived as less intelligent, were feared by others, or faced threats or harassment. Participants were able to specify the perceived reasons for the discrimination they experienced, such as ancestry, gender, race, age, and several other categories, allowing for multiple selections to capture the breadth of discrimination sources. The scale demonstrated excellent reliability in this study (α = .94 and Ω Total = 0.94). The validity of the 10.13039/100004679EDS has been extensively studied and supported, including ethnoracially diverse samples (Lawrence et al., 2022; Lewis et al., 2012).

**Multidimensional Assessment of Parenting Scale (MAPS).** Participants completed the 35-item Revised MAPS (Parent & Forehand, 2017; Rodriguez, Shaffer, & Parent, 2024), which delineates seven distinct parenting dimensions across two overarching categories: positive and negative parenting practices. The positive parenting subscale (α = .68; Ω = 0.69) includes proactive behavior, positive reinforcement, warmth, and supportiveness. Conversely, the negative parenting dimension (α = .70; Ω = 0.73) includes indicators of hostility, lax control, and physical discipline. Each item on this scale is assessed using a 5-point Likert scale, where 1 signifies “never” and 5 denotes “always,” enabling the quantification of parenting behaviors within the identified domains. The validity of the 10.13039/100030385MAPS has been extensively studied and supported, including in parents of ethnoracially diverse backgrounds (Parent & Forehand, 2017; Rodriguez, Shaffer, & Parent, 2024).

**Patient Reported Outcomes Measurement Information System (PROMIS) Pediatric Proxy Report of Pediatric Physical Health.** Parents completed the PROMIS Pediatric Proxy Report, which evaluates six areas: physical functioning, emotional distress, relationships with peers, fatigue, and how pain affects daily activities (Irwin et al., 2012; Varni et al., 2012). This proxy report is derived from existing content domains of the PROMIS pediatric self-report measures. For instance, where the PROMIS pediatric self-report on pain interference asks children if pain affected their sleep ("I had trouble sleeping when I had pain"), the proxy version asks parents to report on their child's sleep difficulties due to pain ("My child had trouble sleeping when he/she had pain"). Responses reflect the past 7 days and utilize a standardized 5-point scale (options range from "never" to "almost always" or from "no trouble" to "not able to do"), or a numeric scale from 0 to 10. In this study, parent-reported subscales for mental (symptoms of anxiety and depression), and physical (e.g., pain, mobility, fatigue, sleep interference) health were analyzed as continuous variables; all six subscales are composed of four items each. Pain interference assesses how pain impacts aspects of daily living such as social interactions, mental focus, emotions, physical activities, and leisure. Mobility evaluates the capacity to perform a variety of physical tasks, from basic movements like rising from bed to more vigorous activities like running. Fatigue gauges the intense feeling of tiredness that hampers the execution of routine tasks, including academic work. Reliability for the subscales was high in this study, with Cronbach's alpha and Omega Total coefficients ranging from 0.83 to 0.92. As with other scales, the validity of the PROMIS has been extensively studied and supported (Mack et al., 2020).

### 2.4 Analytic plan

The analysis began with descriptive statistics to profile the sample's sociodemographic characteristics. We then used sidClustering to categorize all EDS variables into clusters by analyzing 22 variables related to discrimination experiences, including nine items on the frequency of discrimination and thirteen items on perceived reasons for discrimination, such as ancestry, gender, race, age, religion, height, weight, physical appearance, sexual orientation, education, physical disability, skin color, and tribe. This technique, based on a multivariate random forest, assesses variable interactions and calculates data point proximities within the decision trees to identify potential clusters. The sidClustering algorithm automatically calculates proximities based on a data-driven approach, which minimizes human bias in cluster formation. However, human oversight was involved in determining initial parameters, such as the number of variables included. PAM (partitioning about medoids) was used next, based on sidClustering distances, to finalize cluster assignments. The number of clusters was not pre-specified; instead, the optimal number was determined using the Gap statistic with 100 bootstrap samples. This automated process assessed cluster validity by evaluating the ratio of within-to between-cluster distances, balancing model complexity with data fit (Tibshirani et al., 2001). Human oversight was limited to confirming the algorithm's results, ensuring that clusters were interpretable and aligned with our pre-registered hypotheses. For example, clusters were examined for patterns that aligned with known theoretical dimensions of discrimination (e.g., frequency and attributions), with adjustments made only to clarify interpretability if necessary.

Subsequent analyses, which were conducted independently of the clustering analysis, included ANOVAs to evaluate differences in pediatric physical health across clusters, followed by multiple regression analyses to explore interaction effects between discrimination clusters and parenting on pediatric health outcomes. The ANOVAs and regressions were conducted outside the sidClustering analysis to ensure independent validation of the clusters in relation to health outcomes. This separation of clustering from subsequent analyses reduces the risk of overfitting and enhances generalizability. These analyses incorporated interaction terms to assess how parenting might influence pediatric health. Significant interactions were confirmed by adjusting for the false discovery rate (FDR), and adjusted for variables that were significantly different by cluster. A p-value of less than 0.05 was used to denote statistical significance. R version 4.1.1 and packages: randomForestSRC version 2.12.0, cluster version 2.1.2 (Ishwaran, Kogalur, & Kogalur, 2023), gmodels version 2.18.1 (Warnes et al., 2018), and fmsb 0.7.1 (Nakazawa & Nakazawa, 2019) were used to perform analyses. All code used for these analyses, including revised code as a result of requested editorial revisions, is included on the study's OSF page (see Data Availability).

## 3 Results

### 3.1 Sociodemographic characteristics of participants

In this study, a total of 1444 parents participated, with the sample distributed across four clusters. Participants were 32.20% (*n* = 465) non-Hispanic White, 27.15% (*n* = 392) Hispanic, 27.01% (*n* = 390) non-Hispanic Black, and 13.64% (*n* = 197) non-Hispanic Asian. The average age was 39.77 years (SD = 9.74), though this varied significantly by cluster (p < .001), with Cluster 4 having a younger mean age (*M* = 37.22, *SD* = 8.78) compared with other clusters. Most participants were mothers (60.87%; *n* = 879), and the distribution of gender differed across clusters (*p* = .024), with a higher proportion of fathers in Cluster 4 (43.74%) compared to others. Regarding relationship status, 54.99% (*n* = 794) of participants were married, but Cluster 4 had the lowest proportion of married individuals (49.91%) and the highest proportion of never-married individuals (27.34%; *p* = .015). Educational attainment differed across clusters (*p* = .002), with Cluster 4 having the highest percentage of participants with postgraduate degrees (19.58%) but also the highest proportion with only a high school diploma or GED (28.57%). Employment patterns varied (*p* < .001), with Cluster 4 showing the highest proportion of part-time workers (17.28%) but a slightly lower proportion of unemployed individuals (16.23%) compared to other clusters. While household income levels did not significantly differ across clusters (*p* = .135), Cluster 4 had a slightly higher representation of participants earning less than $30,000 annually (22.58%) compared to the total sample. Detailed demographics and differences are presented in Table 1.

**Table 1:** Sociodemographic characteristics of parents (N = 1444).

Characteristic	Cluster 1 (N = 326) M (SD) N (%)	Cluster 2 (N = 368) M (SD) N (%)	Cluster 3 (N = 183) M (SD) N (%)	Cluster 4 (N = 567) M (SD) N (%)	Total M (SD) N (%)	χ^2^, *p*
**Age**	41.32 (10.75)	42.02 (9.52)	40.40 (9.37)	37.22 (8.78)	39.77 (9.74)	23.58, <0.001
**Child Age**	10.41 (4.23)	10.89 (4.45)	11.13 (4.32)	12.98 (59.19)	11.63 (37.24)	0.42, 0.737
**Sex**						9.39, 0.024
Female	201 (61.66)	240 (65.22)	119 (65.03)	319 (56.26)	879 (60.87)	
Male	125 (38.34)	128 (34.78)	64 (34.97)	248 (43.74)	565 (39.13)	
**Ethnoracial Identity**						18.64, 0.028
White/European American	99 (30.37)	116 (31.52)	55 (30.05)	195 (34.39)	465 (32.20)	
Hispanic (all races)	96 (29.45)	91 (24.73)	43 (23.50)	162 (28.57)	392 (27.15)	
Black/African American	80 (24.54)	102 (27.72)	51 (27.87)	157 (27.69)	390 (27.01)	
Asian American/Pacific Islander	51 (15.64)	59 (16.03)	34 (18.58)	53 (9.35)	197 (13.64)	
**Relationship Status**						24.95, 0.015
Never Married	74 (22.70)	66 (17.93)	32 (17.49)	155 (27.34)	327 (22.65)	
Separated or divorced	22 (6.75)	38 (10.33)	17 (9.29)	42 (7.41)	119 (8.24)	
Married	186 (57.06)	216 (58.70)	109 (59.56)	283 (49.91)	794 (54.99)	
In a committed relationship	9 (2.76)	4 (1.09)	2 (1.09)	8 (1.41)	23 (1.59)	
Prefer not to answer	35 (10.74)	44 (11.96)	23 (12.57)	79 (13.93)	181 (12.53)	
**Education**						35.35, 0.002
Post-graduate Degree	55 (16.87)	60 (16.30)	21 (11.48)	111 (19.58)	247 (17.11%)	
Bachelor's Degree	79 (24.23)	117 (31.79%)	56 (30.60)	116 (20.46)	368 (25.48)	
Associate Degree	37 (11.35)	49 (13.32)	32 (17.49)	82 (14.46)	200 (13.85)	
Partial College	54 (16.56)	57 (15.49)	35 (19.13)	88 (15.52)	234 (16.20)	
High School or GED	92 (28.22)	78 (21.20)	34 (18.58)	162 (28.57)	366 (25.35)	
Partial High School	9 (2.76)	7 (1.90)	5 (2.73)	8 (1.41)	29 (2.01)	
**Employment**						26.63, <0.001
Not employed outside the home	84 (25.77)	91 (24.73)	34 (18.58)	92 (16.23)	301 (20.84)	
Work part-time	28 (8.59)	45 (12.23)	21 (11.48)	98 (17.28)	192 (13.30)	
Work full-time	214 (65.64)	232 (63.04)	128 (69.95)	377 (66.49)	951 (65.86)	
**Household Yearly Income (USD)**						24.64, 0.135
Less than $10,000	19 (5.83)	12 (3.26)	14 (7.65)	47 (8.29)	92 (6.37)	
$10,000- $30,000	41 (12.58)	41 (11.14)	20 (10.93)	81 (14.29)	183 (12.67)	
$30,000- $50,000	51 (15.64)	51 (13.86)	29 (15.85)	97 (17.11)	228 (15.79)	
$50,000- $70,000	65 (19.94)	85 (23.10)	29 (15.85)	107 (18.87)	286 (19.81)	
$70,000- $90,000	46 (14.11)	63 (17.12)	26 (14.21)	85 (14.99)	220 (15.24)	
$90,000- $100,000	37 (11.35)	47 (12.77)	23 (12.57)	50 (8.82)	157 (10.87)	
Over $100,000/year	67 (20.55)	69 (18.75)	42 (22.95)	100 (17.64)	278 (19.25)	
Prefer not to answer	19 (5.83)	12 (3.26)	14 (7.65)	47 (8.29)	92 (6.37)	

*Note.* Percentages may not sum to 100% due to rounding.

### 3.2 Determining the optimal number of clusters

Utilizing the Gap statistic to identify the optimal number of clusters, we observed a significant leap in the Gap statistic's value continuing up to four clusters (presented in Supplemental Material), after which the benefits of adding more clusters substantially decreased (Tibshirani et al., 2001). Specifically, transitioning from three to four clusters marked a notable increase of 0.13 in the Gap statistic, indicating a improvement in cluster distinction by incorporating a fourth cluster. In contrast, the transition from four to five clusters showed a minimal increase of 0.06, suggesting that the complexity added by a fifth cluster was not warranted. The model's exceptional performance was further evidenced by an Area Under the Curve (AUC) for the receiver operating characteristic (ROC) = 0.998, surpassing the standard benchmark of 0.90 for excellent predictive accuracy (Fawcett, 2006), alongside a minimal out-of-bag misclassification rate of about 2.98%, indicating high predictive accuracy, based on conventional thresholds indicating good model performance are expected to be <5% (Tibshirani et al., 2001). Most importantly, this four-cluster configuration also aligned with our initial hypotheses.

### 3.3 Labeling discrimination clusters in parents

Analysis revealed a diverse range of discrimination experiences among parents, spanning from negligible to widespread, accompanied by varied attributions. The first cluster, characterized by minimal experiences of discrimination and attributions, was termed "Non-Discriminated/Less Discriminated." The subsequent two clusters, 2 and 3, indicated a spectrum from moderate to significant levels of discrimination, with the discrimination being either broad or targeted. More specifically, Cluster 2, labeled as "Moderately Discriminated with Varied Attributions," included participants reporting sporadic encounters with discriminatory behaviors or attitudes, suggesting intermittent discrimination that was neither pervasive nor constant. Conversely, Cluster 3, described as "Specific and Frequent Discrimination," encompassed reports of regular or constant discrimination, hinting at targeted discrimination linked to specific identities or characteristics. The final cluster, Cluster 4, was recognized as "Highly Discriminated with Broad Attributions" due to reports of frequent discrimination alongside varied attributions, indicating a wide-ranging experience of discrimination. The prevalence of discrimination across clusters is presented in Supplemental Table 2.

### 3.4 Associations between discrimination clusters and pediatric health outcomes

Before testing interactions of discrimination clusters and parenting predicting pediatric health outcomes, we conducted comparisons between main effect models and interaction models to determine whether interaction models significantly improved main effect models. In all cases, interaction models showed a significant improvement over main effects models. As such, unadjusted interaction models are presented and summarized in Table 2, and adjusted interaction models are presented in Table 3. However, Table 3 is presented without adjustments for space and readability; full regression results are presented in Supplemental Table 3. The regression analyses adjusted for sociodemographic variables that were significantly different by cluster, including education, ethnoracial identity, marital status, sex, and employment. Overall, for no significant main effects or interactions with parenting were observed for any outcome for Cluster 2 or Cluster 3 (with the exception of mobility). Positive parenting did not demonstrate significant main effects on anxiety, depression, pain, or sleep. Covariates such as education, employment, marital status, ethnoracial identity, and sex were not significantly associated with the health outcomes examined after adjusting for discrimination and parenting practices. Significant associations are summarized below.

**Table 2:** Unadjusted regression analyses of parent-reported pediatric health outcomes with different clusters of discrimination experience and parenting as predictors.

Predictor	*β*	SE	*t*	*p*	FDR *p*
**Mobility**					

Intercept	0.28	0.05	5.42	<0.001	<0.001
Cluster 2	−0.02	0.07	−0.35	0.730	0.796
Cluster 3	0.11	0.08	1.33	0.182	0.269
Cluster 4	−0.46	0.06	−7.15	<0.001	<0.001
Positive Parenting	0.27	0.04	6.75	<0.001	<0.001
Negative Parenting	−0.08	0.05	−1.52	0.129	0.222
Cluster 2 x Positive	−0.02	0.07	−0.26	0.796	0.796
Cluster 3 x Positive	−0.25	0.08	−3.11	0.002	0.004
Cluster 4 x Positive	0.22	0.05	4.39	<0.001	<0.001
Cluster 2 x Negative	0.02	0.08	0.29	0.769	0.796
Cluster 3 x Negative	0.12	0.10	1.28	0.202	0.269
Cluster 4 x Negative	−0.41	0.06	−6.95	<0.001	<0.001

**Fatigue**					

Intercept	−0.45	0.05	−9.14	<0.001	<0.001
Cluster 2	0.23	0.06	3.56	<0.001	0.001
Cluster 3	0.21	0.08	2.73	0.006	0.011
Cluster 4	0.70	0.06	11.56	<0.001	<0.001
Positive Parenting	−0.09	0.04	−2.28	0.023	0.034
Negative Parenting	0.17	0.05	3.45	0.001	0.001
Cluster 2 x Positive	−0.11	0.06	−1.67	0.096	0.115
Cluster 3 x Positive	−0.03	0.08	−0.46	0.649	0.649
Cluster 4 x Positive	−0.17	0.05	−3.56	<0.001	0.001
Cluster 2 x Negative	0.04	0.07	0.57	0.567	0.619
Cluster 3 x Negative	0.17	0.09	1.88	0.060	0.080
Cluster 4 x Negative	0.46	0.06	8.24	<0.001	<0.001

**Sleep**					

Intercept	−0.47	0.06	−8.20	<0.001	<0.001
Cluster 2	0.33	0.07	4.47	<0.001	<0.001
Cluster 3	0.22	0.09	2.53	0.012	0.023
Cluster 4	0.76	0.07	10.88	<0.001	<0.001
Positive Parenting	0.05	0.04	1.05	0.295	0.443
Negative Parenting	0.20	0.06	3.60	<0.001	0.001
Cluster 2 x Positive	−0.03	0.07	−0.37	0.711	0.772
Cluster 3 x Positive	0.05	0.09	0.53	0.599	0.719
Cluster 4 x Positive	−0.06	0.06	−1.13	0.259	0.443
Cluster 2 x Negative	−0.07	0.08	−0.88	0.380	0.507
Cluster 3 x Negative	0.03	0.10	0.29	0.772	0.772
Cluster 4 x Negative	0.26	0.06	4.06	<0.001	<0.001

**Pain**					

Intercept	−0.40	0.06	−6.98	<0.001	<0.001
Cluster 2	0.21	0.07	2.85	0.004	0.011
Cluster 3	0.20	0.09	2.32	0.021	0.041
Cluster 4	0.66	0.07	9.33	<0.001	<0.001
Positive Parenting	0.00	0.04	0.07	0.948	0.948
Negative Parenting	0.18	0.06	3.19	0.001	0.004
Cluster 2 x Positive	−0.03	0.07	−0.41	0.679	0.766
Cluster 3 x Positive	0.05	0.09	0.53	0.595	0.766
Cluster 4 x Positive	−0.11	0.06	−1.89	0.059	0.101
Cluster 2 x Negative	−0.03	0.08	−0.38	0.702	0.766
Cluster 3 x Negative	−0.04	0.11	−0.40	0.688	0.766
Cluster 4 x Negative	0.27	0.07	4.16	<0.001	<0.001

**Anxiety**					

Intercept	−0.40	0.05	−7.38	<0.001	<0.001
Cluster 2	0.28	0.07	4.00	<0.001	<0.001
Cluster 3	0.18	0.08	2.20	0.028	0.042
Cluster 4	0.66	0.07	9.88	<0.001	<0.001
Positive Parenting	−0.11	0.04	−2.65	0.008	0.016
Negative Parenting	0.20	0.05	3.75	<0.001	<0.001
Cluster 2 x Positive	−0.06	0.07	−0.87	0.386	0.421
Cluster 3 x Positive	−0.03	0.08	−0.38	0.706	0.706
Cluster 4 x Positive	−0.07	0.05	−1.33	0.185	0.222
Cluster 2 x Negative	0.16	0.08	2.00	0.046	0.062
Cluster 3 x Negative	0.23	0.10	2.35	0.019	0.032
Cluster 4 x Negative	0.30	0.06	4.88	<0.001	<0.001

**Depression**					

Intercept	−0.44	0.05	−8.75	<0.001	<0.001
Cluster 2	0.24	0.06	3.65	<0.001	0.001
Cluster 3	0.17	0.08	2.23	0.026	0.039
Cluster 4	0.71	0.06	11.61	<0.001	<0.001
Positive Parenting	−0.08	0.04	−1.99	0.047	0.056
Negative Parenting	0.18	0.05	3.70	<0.001	0.001
Cluster 2 x Positive	−0.14	0.06	−2.17	0.030	0.040
Cluster 3 x Positive	−0.06	0.08	−0.79	0.429	0.429
Cluster 4 x Positive	−0.13	0.05	−2.72	0.007	0.013
Cluster 2 x Negative	0.10	0.07	1.31	0.189	0.207
Cluster 3 x Negative	0.22	0.09	2.47	0.014	0.023
Cluster 4 x Negative	0.43	0.06	7.53	<0.001	<0.001

**Table 3:** Adjusted regression analyses of parent-reported pediatric health outcomes adjusting for sociodemographic predictors.

Predictor	*β*	SE	*t*	*p*	FDR *p*
**Mobility**					

Intercept	3.67	0.21	17.55	<0.001	<0.001
Cluster 2	0.04	0.34	0.11	0.91	0.937
Cluster 3	0.99	0.45	2.2	0.028	0.122
Cluster 4	−0.17	0.26	−0.67	0.505	0.691
Positive Parenting	0.01	0.00	6.14	<0.001	<0.001
Negative Parenting	0.00	0.00	−0.83	0.404	0.679
Cluster 2 x Positive	0.00	0.00	−0.38	0.705	0.873
Cluster 3 x Positive	−0.01	0.00	−2.93	0.003	**.018**
Cluster 4 x Positive	0.01	0.00	3.36	0.001	**.005**
Cluster 2 x Negative	0.00	0.00	0.23	0.816	0.923
Cluster 3 x Negative	0.00	0.00	0.62	0.537	0.698
Cluster 4 x Negative	−0.02	0.00	−6.74	<0.001	**<.001**

**Fatigue**					

Intercept	1.40	0.26	5.41	<0.001	<0.001
Cluster 2	0.73	0.42	1.74	0.083	0.275
Cluster 3	−0.24	0.56	−0.43	0.669	0.829
Cluster 4	0.24	0.32	0.76	0.447	0.727
Positive Parenting	−0.01	0.00	−1.83	0.067	0.275
Negative Parenting	0.01	0.00	2.89	0.004	0.026
Cluster 2 x Positive	−0.01	0.00	−1.56	0.118	0.341
Cluster 3 x Positive	0.00	0.01	−0.3	0.767	0.907
Cluster 4 x Positive	−0.01	0.00	−3.04	0.002	**.021**
Cluster 2 x Negative	0.00	0.00	0.52	0.604	0.785
Cluster 3 x Negative	0.01	0.01	1.93	0.053	0.275
Cluster 4 x Negative	0.03	0.00	7.47	<0.001	<.**001**

**Sleep**					

Intercept	1.50	0.24	6.17	<0.001	<0.001
Cluster 2	0.64	0.39	1.62	0.106	0.457
Cluster 3	−0.18	0.52	−0.35	0.726	0.851
Cluster 4	0.25	0.30	0.84	0.404	0.673
Positive Parenting	0.00	0.00	0.83	0.408	0.673
Negative Parenting	0.01	0.00	3.38	<0.001	0.006
Cluster 2 x Positive	0.00	0.00	−0.24	0.807	0.864
Cluster 3 x Positive	0.00	0.01	0.66	0.512	0.739
Cluster 4 x Positive	0.00	0.00	−0.84	0.399	0.673
Cluster 2 x Negative	0.00	0.00	−1.13	0.26	0.673
Cluster 3 x Negative	0.00	0.01	0.31	0.753	0.851
Cluster 4 x Negative	0.01	0.00	3.72	<0.001	**.003**

**Pain**					

Intercept	1.10	0.32	3.47	<0.001	0.007
Cluster 2	0.86	0.52	1.67	0.095	0.381
Cluster 3	−0.11	0.68	−0.17	0.866	0.917
Cluster 4	0.49	0.39	1.26	0.208	0.602
Positive Parenting	0.00	0.00	0.39	0.699	0.895
Negative Parenting	0.01	0.00	3.06	0.002	0.019
Cluster 2 x Positive	0.00	0.01	−0.70	0.484	0.786
Cluster 3 x Positive	0.01	0.01	0.86	0.391	0.782
Cluster 4 x Positive	−0.01	0.00	−1.63	0.103	0.381
Cluster 2 x Negative	−0.01	0.01	−0.95	0.342	0.740
Cluster 3 x Negative	0.00	0.01	−0.41	0.685	0.895
Cluster 4 x Negative	0.02	0.00	3.62	<0.001	**.007**

**Anxiety**					

Intercept	1.77	0.28	6.38	<0.001	<0.001
Cluster 2	0.08	0.45	0.17	0.866	0.938
Cluster 3	−0.48	0.6	−0.8	0.424	0.727
Cluster 4	0.23	0.34	0.69	0.493	0.727
Positive Parenting	−0.01	0.00	−2.35	0.019	0.123
Negative Parenting	0.01	0.00	2.86	0.004	0.037
Cluster 2 x Positive	0.00	0.00	−0.67	0.503	0.727
Cluster 3 x Positive	0.00	0.01	0.04	0.966	0.966
Cluster 4 x Positive	−0.01	0.00	−1.58	0.114	0.329
Cluster 2 x Negative	0.01	0.00	2.03	0.043	0.186
Cluster 3 x Negative	0.01	0.01	1.83	0.068	0.220
Cluster 4 x Negative	0.02	0.00	4.77	<0.001	**<.001**

**Depression**					

Intercept	1.43	0.26	5.55	<0.001	<0.001
Cluster 2	0.66	0.42	1.58	0.115	0.298
Cluster 3	−0.27	0.55	−0.49	0.621	0.703
Cluster 4	0.21	0.32	0.67	0.501	0.703
Positive Parenting	0.00	0.00	−1.5	0.134	0.317
Negative Parenting	0.01	0.00	2.69	0.007	0.059
Cluster 2 x Positive	−0.01	0.00	−2.06	0.039	0.146
Cluster 3 x Positive	0.00	0.01	−0.68	0.498	0.703
Cluster 4 x Positive	−0.01	0.00	−2.62	0.009	0.059
Cluster 2 x Negative	0.01	0.00	1.71	0.087	0.283
Cluster 3 x Negative	0.01	0.01	2.44	0.015	0.064
Cluster 4 x Negative	0.03	0.00	7.21	<0.001	**<.001**

*Note.* Models controlled for: Education; Marital Status; Ethnoracial Identity; Sex; and Employment. However, these are not presented due to space constraints for ease of readability. Full regression models are presented in Supplemental Materials.

**Mobility.** The interaction between Cluster 3 (Specific and Frequent Discrimination) and positive parenting was significantly associated with reduced mobility, *β* = −0.01, *t* = −2.93, FDR *p* = .018. For Cluster 4, the interaction with negative parenting was associated with reduced mobility, *β* = −0.02, *t* = −6.74, FDR *p* < .001. Conversely, the interaction between Cluster 4 and positive parenting was positively associated with increased mobility, *β* = 0.01, *t* = 3.36, FDR *p* = .005.

**Fatigue.** The interaction between Cluster 4 and negative parenting was significantly associated with higher fatigue levels, *β* = 0.03, *t* = 7.47, FDR *p* < .001. Additionally, the interaction between Cluster 4 and positive parenting was significantly associated with lower fatigue levels, *β* = −0.01, *t* = −3.04, FDR *p* = .021.

**Sleep.** The interaction between Cluster 4 and negative parenting was significantly associated with increased sleep disturbances, *β* = 0.01, *t* = 3.72, FDR *p* = .003.

**Pain.** The interaction between Cluster 4 and negative parenting was significantly associated with higher pain levels, *β* = 0.02, *t* = 3.62, FDR *p* = .007.

**Anxiety.** The interaction between Cluster 4 (Highly Discriminated with Broad Attributions) and negative parenting was significantly associated with increased anxiety symptoms, *β* = 0.02, *t* = 4.77, FDR *p* < .001.

**Depression.** The interaction between Cluster 4 and negative parenting was significantly related to increased depressive symptoms, *β* = 0.03, *t* = 7.21, FDR *p* < .001.

## 4 Discussion

This study, guided by the PSRP Model (Ramey et al., 2015), investigates associations between parental experiences of discrimination, parenting, and pediatric health outcomes. We conceptualize parental discrimination primarily as a stressor that parents experience and that may be associated with their parenting behaviors, and which can, together, be related to worse children's health. The PSRP Model, which emphasizes the influence of parental stressors, including discrimination, on health trajectories, provides a valuable framework for understanding our findings. Further, echoing previous findings (Anderson et al., 2015) our research reaffirms that parental discrimination can be a pervasive stressor that with potential negative implications for child health, particularly in minoritized communities where discrimination is frequent (Carroll, 1998; Coll et al., 1996; McNeil Smith & Landor, 2018). Our results demonstrate that parenting behaviors interact with experiences of discrimination to shape pediatric health, underscoring the importance of context and family dynamics.

Our findings indicate that higher levels of discrimination (as experienced by parents in Cluster 4, “Highly Discriminated with Broad Attributions”) are associated with worse pediatric health outcomes across several dimensions, including mobility, fatigue, sleep disturbances, pain, anxiety, and depression. This supports past theoretical models (Carroll, 1998; Coll et al., 1996; McNeil Smith & Landor, 2018), including the mundane extreme environmental stress model (Carroll, 1998), highlighting the potential effects of discrimination on both mental and physical health. For example, interactions between negative parenting and Cluster 4 were associated with more severe outcomes across most health dimensions, suggesting that negative parenting behaviors may intensify the challenges faced by children in these contexts.

Interactions involving positive parenting revealed mixed results. For mobility, positive parenting was associated with better outcomes in Cluster 4, but this buffering effect was not observed across all health domains. In fact, positive parenting was linked to higher fatigue levels in Cluster 4, suggesting that the protective role of positive parenting may vary depending on the outcome and context. It is possible that discrimination may interfere with parenting and child support. The observed findings between discrimination clusters and parenting, together with existing theoretical models, underscore the intricate interplay between discrimination as a stressor and family factors, emphasizing the need to understand these dynamics to support family and child well-being and resilience.

Interestingly, while negative parenting consistently exacerbated adverse health outcomes in Cluster 4, the interaction between discrimination and parenting behaviors varied in other clusters. This finding highlights the potential buffering role of positive parenting in specific health domains, aligning with prior research emphasizing its protective effects in the context of discrimination (Dotterer & James, 2018; Varner et al., 2018). The consistency of this result across pediatric health outcomes underscores the importance of fostering positive parenting practices to promote resilience in families experiencing high levels of discrimination. On the other hand, negative parenting consistently exacerbates the negative effects of discrimination on child health outcomes. This finding is aligned with the sociocultural family stress model (McNeil Smith & Landor, 2018) which posits that stressors such as discrimination can disrupt family functioning and exacerbate negative parenting, which in turn may adversely affect child health. These results potentially underscore the importance of developing supportive interventions that assist parents from minoritized communities in adopting more positive parenting practices. Such interventions should not only focus on reducing negative parenting but also on enhancing parents' ability to provide warmth and support, particularly in the face of discrimination. Additionally, such interventions may be able to directly support parents themselves, equipping them with tools to better navigate discrimination and its challenges.

Previous studies, such as those by Cobb et al. (2023) have utilized clustering techniques to characterize experiences and attributions of discrimination. These person-centered methods are particularly advantageous over traditional sum and continuous scores approaches, which may fail to capture the full extent of discrimination experienced by individuals with multiple marginalized identities. By focusing on individual patterns rather than aggregate scores, clustering allows for a more granular analysis that honors the complexity of personal experiences. Interestingly, our study successfully replicated a four-cluster model using a distinct population, sample, and analytical method. Traditional methods of analyzing discrimination often rely on aggregated composite scores that focus solely on the summed, average, or aggregated frequency or intensity of discriminatory experiences, overlooking the role of attributions (i.e., perceived reasons for discrimination). By separating frequency and attributions as distinct components, traditional approaches fail to capture their interplay, which is critical for understanding the multidimensional nature of discrimination. Our approach addresses this gap by leveraging sidClustering, an unsupervised machine learning method, to integrate both frequency and attributions into a single, parsimonious analytic framework. This integration allows for an improved examination of how the breadth and frequency of discriminatory experiences compound, offering richer insights into their association with health outcomes. Unlike traditional aggregation, this approach provides a comprehensive view of discrimination's multifaceted impact, enhancing our ability to identify meaningful patterns that may inform targeted interventions for at-risk families. Thus, this replication not only confirms the robustness of clustering approaches but also underscores its utility in revealing patterns of discrimination consistent across diverse settings and populations, thereby enhancing the validity of person-centered analyses in discrimination research.

It is also worth emphasizing the unexpected finding that Cluster 3 did not experience the anticipated adverse effects on child health despite relatively high levels of discrimination merits further exploration. One possible explanation could be the specificity of discrimination experiences within Cluster 3, which might differ qualitatively from the broader, multifaceted, and more frequent discrimination reported in Cluster 4. For instance, targeted discrimination may elicit adaptive coping among parents and children, mitigating its potential health impacts. Alternatively, contextual factors, such as community support, cultural resilience, or differences in perceived discrimination severity, might play a protective role. Further investigation into these contextual variables, as well as longitudinal analyses, may help clarify the mechanisms underlying this finding.

### 4.1 Limitations and future directions

While this study provides valuable insights, it is not without limitations. The cross-sectional design limits our ability to infer causality between the observed variables. Longitudinal studies are needed to better understand the dynamics over time between discrimination, parenting, and child health. Longitudinal data would also facilitate the use of other advanced quantitative approaches, including the potential mechanisms for the identified associations. The use of a paid online survey panel may introduce self-selection bias, as participants opting into such panels may differ from the general population. Monetary incentives could also influence response quality, though quality control measures (e.g., attention checks, response time thresholds) were applied. Future research should replicate these findings with alternative recruitment methods to improve generalizability. Further, demographics such as income and education, may differ from the general population, limiting generalizability. For instance, Cluster 4 participants exhibited higher proportions of postgraduate degrees and lower full-time employment, suggesting that their experiences of discrimination might reflect distinct socioeconomic dynamics. Sociodemographic differences between clusters, such as Cluster 4's higher representation of participants with postgraduate education and part-time employment, may confound the observed relationships. These imbalances highlight the need for future studies to control for and further explore how sociodemographic factors intersect with discrimination and parenting.

Additionally, this study relied on parent-reported measures for both parental discrimination experiences and pediatric health, which introduces the possibility of biases such as social desirability or recall bias. While these measures allowed us to capture parental perspectives on discrimination and its potential impact on their children, future research should consider integrating child self-reports, where age-appropriate, to provide a direct assessment of child health outcomes and their perceptions of family dynamics. Objective health measures, such as medical records or physiological data (e.g., biomarkers of stress) could complement self-reported data and reduce measurement bias.

Similarly, the inclusion of other measures of discrimination, such as observational data, experimental studies, or ecological momentary assessment, could enhance the validity of discrimination measures by reducing reliance on subjective recall. While these enhancements were beyond the scope of the current study, they represent valuable directions for future research seeking to build on our findings.

## 5 Conclusion

This study highlights the effect of parental discrimination on pediatric health outcomes and the moderating role of parenting. We provide an understanding of how discrimination clusters interact with parenting in relation to pediatric health outcomes. A key finding of this study was that only the highest levels of discrimination, as experienced by Cluster 4 (“Highly Discriminated with Broad Attributions”), consistently impacted child health outcomes. These findings suggest that while positive parenting may have a potential mitigating role in certain contexts, it does not uniformly buffer the effects of discrimination. Negative parenting consistently exacerbates adverse outcomes, further emphasizing its detrimental role. The current study contributes to the growing literature by demonstrating the patterns of discrimination effects across different clusters and parenting practices. Future research should replicate these findings using longitudinal and multi-informant designs to clarify causal pathways and interactions. Policy and community-level interventions will be necessary to address the compounding effects of discrimination on pediatric health, particularly among families with multiple marginalized identities. Emphasizing support for positive parenting practices, especially in highly discriminatory contexts, could inform targeted interventions to promote better health outcomes for children and families. While support for better parenting may be critical, it must be complemented by systemic interventions aimed at reducing discrimination itself. Addressing structural inequities and societal biases that perpetuate discrimination is essential to creating an environment where families can thrive. Together, individual, community, and systemic approaches will be necessary to promote better health outcomes for children and families.

## CRediT authorship contribution statement

**Violeta J. Rodriguez:** Writing – review & editing, Writing – original draft, Methodology, Investigation, Funding acquisition, Formal analysis, Conceptualization. **Dominique L. La Barrie:** Writing – review & editing.

## Ethical statement

This study was conducted in accordance with the ethical principles outlined in the Declaration of Helsinki and received approval from the University of Illinois Urbana Champaign. Informed consent was obtained from all participants prior to their involvement in the study. All data collected were handled confidentially and stored securely to protect participant privacy. Participants were informed of their right to withdraw from the study at any time without any consequences. The study adhered to all relevant guidelines and regulations to ensure the ethical treatment of participants.

## Funding

This research was supported by a NIH Director's Early Independence Award (DP5-OD036508) awarded to Violeta J. Rodriguez.

## Declaration of competing intrest

We have no conflict of interest to disclose.

## Data Availability

**Preregistration:** The current study was preregistered via the OSF, and the data, R analytic code, and additional appendices are available on the study’s OSF page (https://osf.io/yz4ex/?view_only=16af93e7375e41efba7e4ac7a7d0b24f).
