# Supplementary material for: Parent discrimination clusters and pediatric health in a national survey: The modifying effect of parenting
Source: SSM Popul Health. 2025 Jan 25;29:101757. doi: 10.1016/j.ssmph.2025.101757 (PMC11872493; doi:10.1016/j.ssmph.2025.101757)
Supplement: Multimedia component 1 [file mmc1.docx]

**Supplemental Materials**

**Supplemental Table 1**
**Gap Statistic for Cluster Analysis**

| **k** | **logW** | **E(logW)** | **Gap** | **SE(sim)** |
| --- | --- | --- | --- | --- |
| 1 | 8.23 | 8.24 | 0.01 | 0.006 |
| 2 | 7.56 | 7.99 | 0.42 | 0.005 |
| 3 | 7.30 | 7.91 | 0.61 | 0.005 |
| 4 | 7.12 | 7.89 | 0.77 | 0.004 |
| 5 | 7.04 | 7.87 | 0.83 | 0.005 |

**Note**. *k* represents the number of clusters. logW is the logarithm of within-cluster sum of squares, E(logW) is the expected value of logW under the null hypothesis, Gap is the difference between logW and E(logW), and SE(sim) is the standard error of the simulation.

**Supplemental Figure 1**
**Gap Statistic for Determining Optimal Number of Clusters**


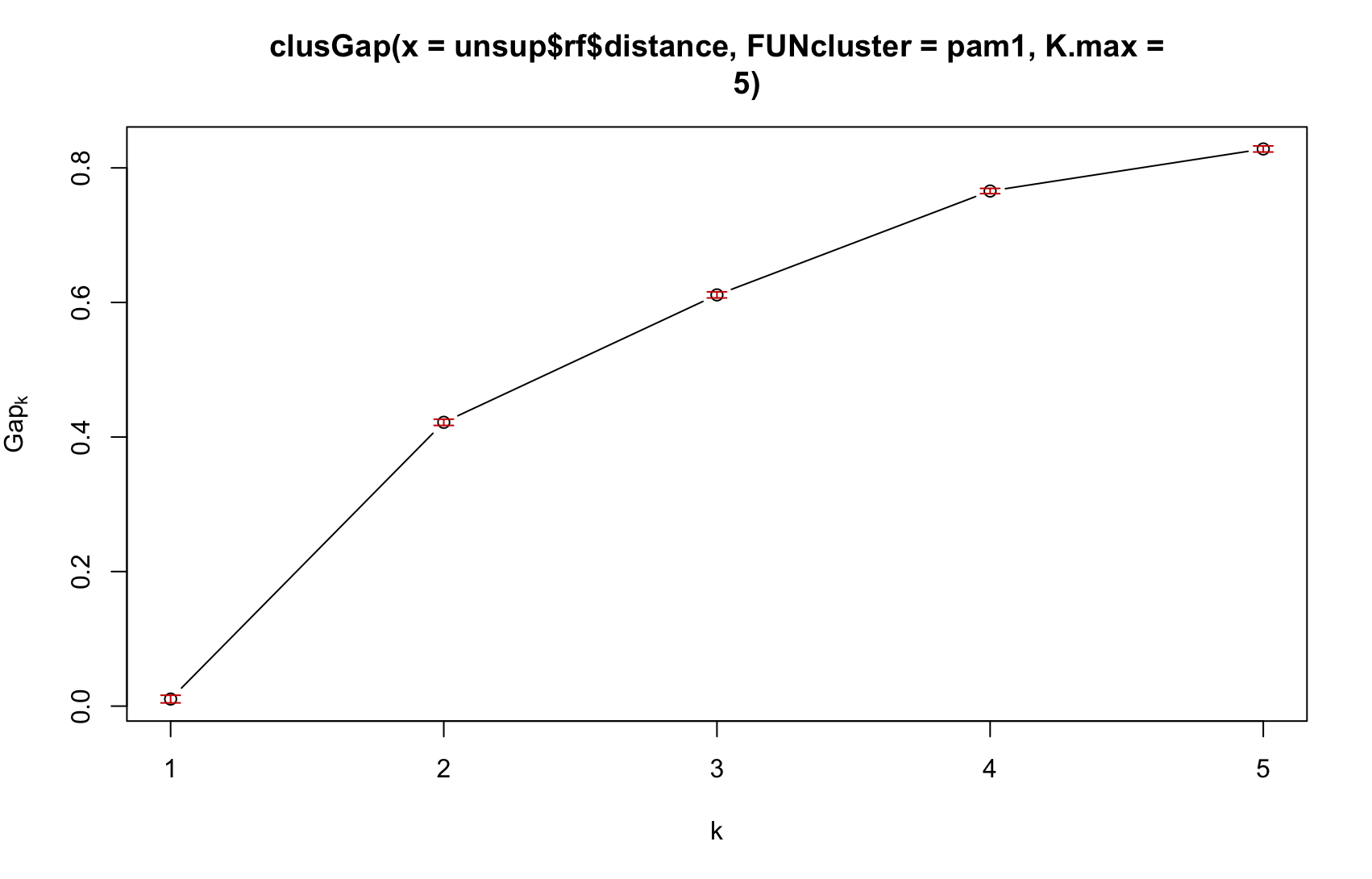


**Legend:**
The plot shows the Gap statistic (Gapkk​) across different numbers of clusters (*k*), calculated using the unsupervised random forest distance matrix. Error bars represent the standard error of simulation (**SE(sim)**).

**Note.** The Gap statistic compares the log of within-cluster dispersion to its expected value under a null distribution (uniformly distributed reference data). Higher Gap values indicate better-defined clusters. While *k* = 5 is optimal based on the firstSEmax method, *k* = 4 still demonstrates a substantial gap, suggesting it may also be a viable choice depending on practical considerations and hypotheses consistent with out pre-registration.

**Supplemental Table 2.**

Counts and Proportions for Experiences of Discrimination by Cluster *(N = 1444)*

| Description | **Cluster 1 (N=326)**  **N (%)** | **Cluster 2 (N=368)**  **N (%)** | **Cluster 3 (N=183)**  **N (%)** | **Cluster 4 (N=567)**  **N (%)** | **χ^2^, *p*** |
| --- | --- | --- | --- | --- | --- |
| Less Courtesy (0) | 326 (100) | 60 (16.30) | 145 (79.23) | 29 (5.11) | 664.67, < .001 |
| 1 | 0 (0) | 158 (42.93) | 26 (14.21) | 37 (6.53) |  |
| 2 | 0 (0) | 115 (31.25) | 5 (2.73) | 142 (25.04) |  |
| 3 | 0 (0) | 19 (5.16) | 2 (1.09) | 163 (28.75) |  |
| 4 | 0 (0) | 8 (2.17) | 4 (2.19) | 103 (18.17) |  |
| 5 | 0 (0) | 8 (2.17) | 1 (0.55) | 93 (16.40) |  |
| Less Respect (0) | 326 (100) | 49 (13.32) | 175 (95.63) | 26 (4.59) | 634.51, < .001 |
| 1 | 0 (0) | 164 (44.57) | 8 (4.37) | 60 (10.58) |  |
| 2 | 0 (0) | 119 (32.34) | 0 (0) | 143 (25.22) |  |
| 3 | 0 (0) | 17 (4.62) | 0 (0) | 148 (26.10) |  |
| 4 | 0 (0) | 7 (1.90) | 0 (0) | 112 (19.75) |  |
| 5 | 0 (0) | 12 (3.26) | 0 (0) | 78 (13.76) |  |
| Poorer Service (0) | 326 (100) | 125 (33.97) | 137 (74.86) | 47 (8.29) | 536.44, < .001 |
| 1 | 0 (0) | 160 (43.48) | 34 (18.58) | 55 (9.70) |  |
| 2 | 0 (0) | 75 (20.38) | 9 (4.92) | 175 (30.86) |  |
| 3 | 0 (0) | 1 (0.27) | 1 (0.55) | 154 (27.16) |  |
| 4 | 0 (0) | 4 (1.09) | 0 (0) | 80 (14.11) |  |
| 5 | 0 (0) | 3 (0.82) | 2 (1.09) | 56 (9.88) |  |
| Think Not Smart (0) | 326 (100) | 119 (32.34) | 148 (80.87) | 25 (4.41) | 562.77, < .001 |
| 1 | 0 (0) | 139 (37.77) | 27 (14.75) | 46 (8.11) |  |
| 2 | 0 (0) | 88 (23.91) | 6 (3.28) | 150 (26.46) |  |
| 3 | 0 (0) | 12 (3.26) | 2 (1.09) | 136 (23.99) |  |
| 4 | 0 (0) | 5 (1.36) | 0 (0) | 117 (20.63) |  |
| 5 | 0 (0) | 5 (1.36) | 0 (0) | 93 (16.40) |  |
| People Act Afraid (0) | 326 (100) | 226 (61.41) | 147 (80.33) | 103 (18.17) | 337.06, < .001 |
| 1 | 0 (0) | 83 (22.55) | 19 (10.38) | 69 (12.17) |  |
| 2 | 0 (0) | 40 (10.87) | 7 (3.83) | 128 (22.57) |  |
| 3 | 0 (0) | 8 (2.17) | 8 (4.37) | 118 (20.81) |  |
| 4 | 0 (0) | 6 (1.63) | 1 (0.55) | 95 (16.75) |  |
| 5 | 0 (0) | 5 (1.36) | 1 (0.55) | 54 (9.52) |  |
| Think Dishonest (0) | 326 (100) | 232 (63.04) | 163 (89.07) | 77 (13.58) | 340.86, < .001 |
| 1 | 0 (0) | 111 (30.16) | 15 (8.20) | 66 (11.64) |  |
| 2 | 0 (0) | 23 (6.25) | 4 (2.19) | 122 (21.52) |  |
| 3 | 0 (0) | 1 (0.27) | 1 (0.55) | 121 (21.34) |  |
| 4 | 0 (0) | 1 (0.27) | 0 (0) | 106 (18.69) |  |
| 5 | 0 (0) | 0 (0) | 0 (0) | 75 (13.23) |  |
| Act Better Than (0) | 326 (100) | 70 (19.02) | 74 (40.44) | 20 (3.53) | 819.78, < .001 |
| 1 | 0 (0) | 117 (31.79) | 54 (29.51) | 41 (7.23) |  |
| 2 | 0 (0) | 132 (35.87) | 40 (21.86) | 137 (24.16) |  |
| 3 | 0 (0) | 35 (9.51) | 9 (4.92) | 142 (25.04) |  |
| 4 | 0 (0) | 5 (1.36) | 2 (1.09) | 104 (18.34) |  |
| 5 | 0 (0) | 9 (2.45) | 4 (2.19) | 123 (21.69) |  |
| Insulted (0) | 326 (100) | 206 (55.98) | 149 (81.42) | 65 (11.46) | 393.97, < .001 |
| 1 | 0 (0) | 123 (33.42) | 24 (13.11) | 73 (12.87) |  |
| 2 | 0 (0) | 29 (7.88) | 8 (4.37) | 124 (21.87) |  |
| 3 | 0 (0) | 8 (2.17) | 1 (0.55) | 128 (22.57) |  |
| 4 | 0 (0) | 2 (0.54) | 1 (0.55) | 119 (20.99) |  |
| 5 | 0 (0) | 0 (0) | 0 (0) | 58 (10.23) |  |
| Threatened or Harassed (0) | 326 (100) | 267 (72.55) | 163 (89.07) | 108 (19.05) | 282.66, <.001 |
| 1 | 0 (0) | 86 (23.37) | 14 (7.65) | 97 (17.11) |  |
| 2 | 0 (0) | 14 (3.80) | 3 (1.64) | 125 (22.05) |  |
| 3 | 0 (0) | 1 (0.27) | 1 (0.55) | 102 (17.99) |  |
| 4 | 0 (0) | 0 (0) | 0 (0) | 74 (13.05) |  |
| 5 | 0 (0) | 0 (0) | 2 (1.09) | 61 (10.76) |  |

*Note.* Percentages may not sum to 100% due to rounding.

**Supplemental Table 3.**

Regression Analyses of Parent-Reported Pediatric Health Outcomes Adjusting for Sociodemographic Predictors

| Predictor | ***β*** | **SE** | ***t*** | ***p*** | **FDR *p*** |
| --- | --- | --- | --- | --- | --- |
| **Anxiety** | | | | | |
| Intercept | 1.77 | 0.28 | 6.38 | < .001 | < .001 |
| Cluster 2 | 0.08 | 0.45 | 0.17 | 0.866 | 0.938 |
| Cluster 2 x Negative | 0.01 | 0 | 2.03 | 0.043 | 0.186 |
| Cluster 2 x Positive | 0 | 0 | -0.67 | 0.503 | 0.727 |
| Cluster 3 | -0.48 | 0.6 | -0.8 | 0.424 | 0.727 |
| Cluster 3 x Negative | 0.01 | 0.01 | 1.83 | 0.068 | 0.22 |
| Cluster 3 x Positive | 0 | 0.01 | 0.04 | 0.966 | 0.966 |
| Cluster 4 | 0.23 | 0.34 | 0.69 | 0.493 | 0.727 |
| Cluster 4 x Negative | 0.02 | 0 | 4.77 | < .001 | **< .001** |
| Cluster 4 x Positive | -0.01 | 0 | -1.58 | 0.114 | 0.329 |
| Education Associate Degree | 0.08 | 0.07 | 1.11 | 0.266 | 0.576 |
| Education Bachelor's Degree | 0.12 | 0.08 | 1.47 | 0.141 | 0.334 |
| Education Partial College | -0.17 | 0.17 | -1 | 0.316 | 0.588 |
| Education Partial High School | 0.04 | 0.08 | 0.46 | 0.646 | 0.816 |
| Education Post-graduate Degree | -0.05 | 0.08 | -0.7 | 0.482 | 0.727 |
| Ethnoracial Asian American/Pacific Islander | -0.08 | 0.08 | -1.03 | 0.302 | 0.588 |
| Ethnoracial Black/African American | -0.14 | 0.06 | -2.19 | 0.029 | 0.151 |
| Ethnoracial Hispanic (all races) | -0.03 | 0.06 | -0.44 | 0.659 | 0.816 |
| Marital Status Committed Relationship | -0.01 | 0.18 | -0.07 | 0.945 | 0.966 |
| Marital Status Married | -0.02 | 0.06 | -0.32 | 0.749 | 0.847 |
| Marital Status Separated/Divorced | 0.17 | 0.09 | 1.86 | 0.064 | 0.22 |
| Negative Parenting | 0.01 | 0 | 2.86 | 0.004 | 0.037 |
| Sex Male | 0.02 | 0.05 | 0.5 | 0.614 | 0.816 |
| Positive Parenting | -0.01 | 0 | -2.35 | 0.019 | 0.123 |
| Work full-time | 0.09 | 0.06 | 1.49 | 0.137 | 0.334 |
| Work part-time | 0.03 | 0.08 | 0.36 | 0.716 | 0.846 |
| **Depression** |  |  |  |  |  |
| Intercept | 1.43 | 0.26 | 5.55 | < .001 | < .001 |
| Cluster 2 | 0.66 | 0.42 | 1.58 | 0.115 | 0.298 |
| Cluster 2 x Negative | 0.01 | 0 | 1.71 | 0.087 | 0.283 |
| Cluster 2 x Positive | -0.01 | 0 | -2.06 | 0.039 | 0.146 |
| Cluster 3 | -0.27 | 0.55 | -0.49 | 0.621 | 0.703 |
| Cluster 3 x Negative | 0.01 | 0.01 | 2.44 | 0.015 | 0.064 |
| Cluster 3 x Positive | 0 | 0.01 | -0.68 | 0.498 | 0.703 |
| Cluster 4 | 0.21 | 0.32 | 0.67 | 0.501 | 0.703 |
| Cluster 4 x Negative | 0.03 | 0 | 7.21 | < .001 | **< .001** |
| Cluster 4 x Positive | -0.01 | 0 | -2.62 | 0.009 | 0.059 |
| Education Associate Degree | 0.04 | 0.06 | 0.61 | 0.542 | 0.703 |
| Education Bachelor's Degree | 0.09 | 0.07 | 1.25 | 0.213 | 0.462 |
| Education Partial College | -0.02 | 0.16 | -0.12 | 0.904 | 0.934 |
| Education Partial High School | 0.03 | 0.07 | 0.37 | 0.711 | 0.77 |
| Education Post-graduate Degree | -0.11 | 0.07 | -1.6 | 0.11 | 0.298 |
| Ethnoracial Asian American/Pacific Islander | -0.04 | 0.07 | -0.59 | 0.555 | 0.703 |
| Ethnoracial Black/African American | -0.15 | 0.06 | -2.49 | 0.013 | 0.064 |
| Ethnoracial Hispanic (all races) | -0.06 | 0.06 | -1.01 | 0.313 | 0.581 |
| Marital Status Committed Relationship | -0.14 | 0.16 | -0.85 | 0.395 | 0.641 |
| Marital Status Married | -0.03 | 0.06 | -0.51 | 0.613 | 0.703 |
| Marital Status Separated/Divorced | 0.08 | 0.08 | 0.93 | 0.354 | 0.613 |
| Negative Parenting | 0.01 | 0 | 2.69 | 0.007 | 0.059 |
| Sex Male | -0.02 | 0.05 | -0.53 | 0.596 | 0.703 |
| Positive Parenting | 0 | 0 | -1.5 | 0.134 | 0.317 |
| Work full-time | 0.06 | 0.06 | 1.04 | 0.299 | 0.581 |
| Work part-time | -0.01 | 0.08 | -0.08 | 0.934 | 0.934 |
| **Fatigue** | | | | | |
| Intercept | 1.4 | 0.26 | 5.41 | < .001 | < .001 |
| Cluster 2 | 0.73 | 0.42 | 1.74 | 0.083 | 0.275 |
| Cluster 2 x Negative | 0 | 0 | 0.52 | 0.604 | 0.785 |
| Cluster 2 x Positive | -0.01 | 0 | -1.56 | 0.118 | 0.341 |
| Cluster 3 | -0.24 | 0.56 | -0.43 | 0.669 | 0.829 |
| Cluster 3 x Negative | 0.01 | 0.01 | 1.93 | 0.053 | 0.275 |
| Cluster 3 x Positive | 0 | 0.01 | -0.3 | 0.767 | 0.907 |
| Cluster 4 | 0.24 | 0.32 | 0.76 | 0.447 | 0.727 |
| Cluster 4 x Negative | 0.03 | 0 | 7.47 | < .001 | **< .001** |
| Cluster 4 x Positive | -0.01 | 0 | -3.04 | 0.002 | **0.021** |
| Education Associate Degree | -0.06 | 0.06 | -1 | 0.316 | 0.547 |
| Education Bachelor's Degree | 0.05 | 0.07 | 0.64 | 0.525 | 0.785 |
| Education Partial College | -0.09 | 0.16 | -0.55 | 0.584 | 0.785 |
| Education Partial High School | 0.01 | 0.07 | 0.15 | 0.883 | 0.941 |
| Education Post-graduate Degree | -0.1 | 0.07 | -1.44 | 0.149 | 0.353 |
| Ethnoracial Asian American/Pacific Islander | 0.01 | 0.07 | 0.16 | 0.873 | 0.941 |
| Ethnoracial Black/African American | -0.1 | 0.06 | -1.73 | 0.085 | 0.275 |
| Ethnoracial Hispanic (all races) | -0.07 | 0.06 | -1.19 | 0.235 | 0.493 |
| Marital Status Committed Relationship | -0.1 | 0.17 | -0.6 | 0.548 | 0.785 |
| Marital Status Married | -0.01 | 0.06 | -0.12 | 0.905 | 0.941 |
| Marital Status Separated/Divorced | 0.09 | 0.08 | 1.12 | 0.264 | 0.493 |
| Negative Parenting | 0.01 | 0 | 2.89 | 0.004 | 0.026 |
| Sex Male | 0 | 0.05 | -0.03 | 0.975 | 0.975 |
| Positive Parenting | -0.01 | 0 | -1.83 | 0.067 | 0.275 |
| Work full-time | 0.09 | 0.06 | 1.49 | 0.136 | 0.353 |
| Work part-time | 0.09 | 0.08 | 1.11 | 0.266 | 0.493 |
| **Mobility** | | | | | |
| Intercept | 3.67 | 0.21 | 17.55 | < .001 | < .001 |
| Cluster 2 | 0.04 | 0.34 | 0.11 | 0.91 | 0.937 |
| Cluster 2 x Negative | 0 | 0 | 0.23 | 0.816 | 0.923 |
| Cluster 2 x Positive | 0 | 0 | -0.38 | 0.705 | 0.873 |
| Cluster 3 | 0.99 | 0.45 | 2.2 | 0.028 | 0.122 |
| Cluster 3 x Negative | 0 | 0 | 0.62 | 0.537 | 0.698 |
| Cluster 3 x Positive | -0.01 | 0 | -2.93 | 0.003 | **0.018** |
| Cluster 4 | -0.17 | 0.26 | -0.67 | 0.505 | 0.691 |
| Cluster 4 x Negative | -0.02 | 0 | -6.74 | < .001 | **< .001** |
| Cluster 4 x Positive | 0.01 | 0 | 3.36 | < .001 | **0.005** |
| Education Associate Degree | 0 | 0.05 | 0.08 | 0.937 | 0.937 |
| Education Bachelor's Degree | -0.04 | 0.06 | -0.67 | 0.501 | 0.691 |
| Education Partial College | -0.12 | 0.13 | -0.95 | 0.343 | 0.636 |
| Education Partial High School | -0.08 | 0.06 | -1.39 | 0.164 | 0.474 |
| Education Post-graduate Degree | 0.09 | 0.06 | 1.57 | 0.117 | 0.382 |
| Ethnoracial Asian American/Pacific Islander | 0.13 | 0.06 | 2.13 | 0.034 | 0.125 |
| Ethnoracial Black/African American | 0.01 | 0.05 | 0.16 | 0.876 | 0.937 |
| Ethnoracial Hispanic (all races) | 0.04 | 0.05 | 0.81 | 0.418 | 0.679 |
| Marital Status Committed Relationship | 0.13 | 0.13 | 0.99 | 0.323 | 0.636 |
| Marital Status Married | 0.06 | 0.05 | 1.33 | 0.185 | 0.48 |
| Marital Status Separated/Divorced | 0.07 | 0.07 | 1.08 | 0.279 | 0.636 |
| Negative Parenting | 0 | 0 | -0.83 | 0.404 | 0.679 |
| Sex Male | -0.03 | 0.04 | -0.69 | 0.49 | 0.691 |
| Positive Parenting | 0.01 | 0 | 6.14 | < .001 | < .001 |
| Work full-time | -0.05 | 0.05 | -0.96 | 0.339 | 0.636 |
| Work part-time | -0.02 | 0.06 | -0.24 | 0.809 | 0.923 |
| **Pain** | | | | | |
| Intercept | 1.1 | 0.32 | 3.47 | < .001 | 0.007 |
| Cluster 2 | 0.86 | 0.52 | 1.67 | 0.095 | 0.381 |
| Cluster 2 x Negative | -0.01 | 0.01 | -0.95 | 0.342 | 0.74 |
| Cluster 2 x Positive | 0 | 0.01 | -0.7 | 0.484 | 0.786 |
| Cluster 3 | -0.11 | 0.68 | -0.17 | 0.866 | 0.917 |
| Cluster 3 x Negative | 0 | 0.01 | -0.41 | 0.685 | 0.895 |
| Cluster 3 x Positive | 0.01 | 0.01 | 0.86 | 0.391 | 0.782 |
| Cluster 4 | 0.49 | 0.39 | 1.26 | 0.208 | 0.602 |
| Cluster 4 x Negative | 0.02 | 0 | 3.62 | < .001 | **0.007** |
| Cluster 4 x Positive | -0.01 | 0 | -1.63 | 0.103 | 0.381 |
| Education Associate Degree | -0.16 | 0.08 | -1.97 | 0.049 | 0.319 |
| Education Bachelor's Degree | 0.15 | 0.09 | 1.69 | 0.091 | 0.381 |
| Education Partial College | -0.06 | 0.19 | -0.33 | 0.745 | 0.895 |
| Education Partial High School | -0.07 | 0.09 | -0.74 | 0.461 | 0.786 |
| Education Post-graduate Degree | -0.12 | 0.09 | -1.33 | 0.182 | 0.593 |
| Ethnoracial Asian American/Pacific Islander | -0.01 | 0.09 | -0.1 | 0.917 | 0.917 |
| Ethnoracial Black/African American | -0.04 | 0.07 | -0.55 | 0.582 | 0.883 |
| Ethnoracial Hispanic (all races) | -0.08 | 0.07 | -1.09 | 0.274 | 0.648 |
| Marital Status Committed Relationship | -0.02 | 0.2 | -0.12 | 0.905 | 0.917 |
| Marital Status Married | 0.02 | 0.07 | 0.26 | 0.792 | 0.895 |
| Marital Status Separated/Divorced | 0.08 | 0.1 | 0.78 | 0.437 | 0.786 |
| Negative Parenting | 0.01 | 0 | 3.06 | 0.002 | 0.019 |
| Sex Male | 0.06 | 0.06 | 1.16 | 0.247 | 0.643 |
| Positive Parenting | 0 | 0 | 0.39 | 0.699 | 0.895 |
| Work full-time | -0.02 | 0.07 | -0.28 | 0.783 | 0.895 |
| Work part-time | -0.05 | 0.1 | -0.51 | 0.612 | 0.883 |
| **Sleep** | | | | | |
| Intercept | 1.5 | 0.24 | 6.17 | < .001 | < .001 |
| Cluster 2 | 0.64 | 0.39 | 1.62 | 0.106 | 0.457 |
| Cluster 2 x Negative | 0 | 0 | -1.13 | 0.260 | 0.673 |
| Cluster 2 x Positive | 0 | 0 | -0.24 | 0.807 | 0.864 |
| Cluster 3 | -0.18 | 0.52 | -0.35 | 0.726 | 0.851 |
| Cluster 3 x Negative | 0 | 0.01 | 0.31 | 0.753 | 0.851 |
| Cluster 3 x Positive | 0 | 0.01 | 0.66 | 0.512 | 0.739 |
| Cluster 4 | 0.25 | 0.3 | 0.84 | 0.404 | 0.673 |
| Cluster 4 x Negative | 0.01 | 0 | 3.72 | < .001 | **0.003** |
| Cluster 4 x Positive | 0 | 0 | -0.84 | 0.399 | 0.673 |
| Education Associate Degree | 0.03 | 0.06 | 0.51 | 0.612 | 0.837 |
| Education Bachelor's Degree | 0.09 | 0.07 | 1.35 | 0.179 | 0.629 |
| Education Partial College | -0.1 | 0.15 | -0.7 | 0.483 | 0.739 |
| Education Partial High School | 0.03 | 0.07 | 0.43 | 0.669 | 0.851 |
| Education Post-graduate Degree | 0.05 | 0.07 | 0.82 | 0.414 | 0.673 |
| Ethnoracial Asian American/Pacific Islander | -0.06 | 0.07 | -0.89 | 0.375 | 0.673 |
| Ethnoracial Black/African American | -0.09 | 0.06 | -1.65 | 0.100 | 0.457 |
| Ethnoracial Hispanic (all races) | -0.01 | 0.05 | -0.15 | 0.883 | 0.883 |
| Marital Status Committed Relationship | 0.05 | 0.16 | 0.35 | 0.730 | 0.851 |
| Marital Status Married | -0.01 | 0.05 | -0.21 | 0.831 | 0.864 |
| Marital Status Separated/Divorced | 0.08 | 0.08 | 1 | 0.318 | 0.673 |
| Negative Parenting | 0.01 | 0 | 3.38 | < .001 | 0.006 |
| Sex Male | -0.09 | 0.04 | -2.2 | 0.028 | 0.184 |
| Positive Parenting | 0 | 0 | 0.83 | 0.408 | 0.673 |
| Work full-time | -0.07 | 0.06 | -1.3 | 0.194 | 0.629 |
| Work part-time | -0.08 | 0.07 | -1.09 | 0.276 | 0.673 |

*Note.* Reference groups for the categorical predictors are as follows: Education: High School or GED; Marital Status: Never Married; Ethnoracial Identity: White/European American; Sex: Female; Employment: Not employed outside the home.
